# Genetic anchoring of whole-genome shotgun assemblies

**DOI:** 10.3389/fgene.2014.00208

**Published:** 2014-07-07

**Authors:** Martin Mascher, Nils Stein

**Affiliations:** Leibniz Institute of Plant Genetics and Crop Plant Research, Stadt SeelandGermany

**Keywords:** next-generation sequencing, whole-genome shotgun assembly, assembly anchoring, genetic mapping, genotyping-by-sequencing, single-nucleotide polymorphisms, mapping populations

## Abstract

The recent advances in sequencing throughput and genome assembly algorithms have established whole-genome shotgun (WGS) assemblies as the cornerstone of the genomic infrastructure for many species. WGS assemblies can be constructed with comparative ease and give a comprehensive representation of the gene space even of large and complex genomes. One major obstacle in utilizing WGS assemblies for important research applications such as gene isolation or comparative genomics has been the lack of chromosomal positioning and contextualization of short sequence contigs. Assigning chromosomal locations to sequence contigs required the construction and integration of genome-wide physical maps and dense genetic linkage maps as well as synteny to model species. Recently, methods to rapidly construct ultra-dense linkage maps encompassing millions of genetic markers from WGS sequencing data of segregating populations have made possible the direct assignment of genetic positions to short sequence contigs. Here, we review recent developments in the integration of WGS assemblies and sequence-based linkage maps, discuss challenges for further improvement of the methodology and outline possible applications building on genetically anchored WGS assemblies.

## INTRODUCTION

Next-generation sequencing (NGS) has facilitated the rapid collection of vast amounts of genomic sequence data, enabling whole-genome shotgun (WGS) assemblies in species with huge genomes (Li et al., 2010; Jia et al., 2013; Ling et al., 2013; Nystedt et al., 2013). Compared with approaches based on physical maps, WGS assemblies are rapidly made, are comparatively cheap and represent an easy way to gain a comprehensive view of the gene complement of a species, even for species without prior availability of genomic resources. Nevertheless, *de novo* sequence assembly from short sequence reads remains a formidable algorithmic challenge requiring large amounts of sequence data and powerful compute resources. A recent comparative benchmarking (Bradnam et al., 2013) of assembly pipelines on real datasets highlighted substantial differences in the performance of different algorithmic approaches. The main limitation of WGS assemblies for downstream applications is their fragmentation (Green, 1997): they often consist of up to millions of short contiguous pieces of sequence (contigs), which may be grouped and partially ordered by long-distance mate-pair reads to form scaffolds.

The primary algorithmic challenge of sequence assembly – and thus the origin of the fragmentation – are repeat elements (Alkan et al., 2011b), whose numerous copies are nearly identical, are difficult to resolve with short NGS reads and thus tend to be assembled into a single collapsed sequence contig. Moreover, contigs representing single-copy regions cannot be unambiguously extended at the border of repetitive elements and terminate there. The lack of contiguity of WGS assemblies is a major impediment to downstream analyses. Sequence-based high-throughput genotyping and its applications such as genome-wide association or population genetic studies rely on the visualization of features [single-nucleotide polymorphisms (SNPs), peaks of summary statistics] along the chromosomes, often applying sliding-windows to aggregate the information of neighboring contigs (Luikart et al., 2003; Schneeberger et al., 2009; Andrews and Luikart, 2014; Ellegren, 2014). Without any notion of order or vicinity of contigs, such approaches are impossible.

The process of assigning chromosomal locations to contigs of an assembly is referred to as anchoring. The ultimate goal of this process is to establish pseudomolecules, single accurately ordered sequence scaffolds for each chromosome with as little intervening gaps as possible. Lacking in completeness – in particular in the repetitive portion of the genome – and contiguity, WGS assemblies of large and complex genomes of flowering plants or mammals have so far not attained the quality of a draft genome (Alkan et al., 2011b; Feuillet et al., 2011). High-quality reference sequences continue to be constructed with the help of physical maps and sequencing single bacterial artificial chromosomes (BACs; Groenen et al., 2012; Amborella Genome Project, 2013). However, this hierarchical shotgun approach entails the laborious and expensive steps of BAC library construction, finger-printing and clone-by-clone sequencing (Ariyadasa and Stein, 2012).

If extensive physical mapping resources are not available (as is the case for many non-model species), reference genomes of related species may serve as proxy to order WGS assemblies, but approaches based on genome collinearity (Mayer et al., 2009) are restricted to genic regions and their accuracy is bounded by the degree of syntenic conservation between related species. Recent translocations or duplications of single genes or larger genomic regions may reduce interspecific collinearity and thus impact the accuracy of synteny-guided assembly ordering (Wicker et al., 2011). This approach is also limited to the gene-space, as intergenic, repetitive sequences evolve very fast and show little conservation even between individuals of a single species (Brunner et al., 2005). It is therefore desirable to have methods at hand that can provide fast and cost-efficient access to an at least partially ordered WGS assembly.

## GENETIC ANCHORING OF WGS ASSEMBLIES

For more than a century, genetic mapping has been a universal method to order genomic loci along the chromosomes of sexually reproducing species. Theoretical models of allelic segregation in experimental mapping populations have long been established (Morgan et al., 1922) and various algorithms applying these principles to construct genetic linkage maps from genotypic data have been implemented [reviewed in Cheema and Dicks (2009)]. During the last decades, advances in genetic mapping have been concomitant with the development of molecular marker technologies (Henry, 2012). NGS-based genotyping has recently enabled the simultaneous and near-exhaustive assay of every sequence polymorphism segregating in a mapping population (Huang et al., 2009; Davey et al., 2011). Genotyping-by-sequencing of mapping populations has first been employed in species with high-quality map-based reference sequences such as rice (Huang et al., 2009; Xie et al., 2010) or *Drosophila* (Andolfatto et al., 2011). This obviated the need for inferring marker order *de novo* from the genotypic data and enabled the efficient elimination of missing data through a sliding-window approach (Xie et al., 2010).

Because whole genome resequencing is still too expensive to deeply sequence a large number of individuals of species with large genomes, methods have been designed that reduce the genomic complexity either by restriction enzyme digestion (Altshuler et al., 2000; Baird et al., 2008; Elshire et al., 2011) or sequence capture with oligonucleotide baits (Hodges et al., 2007; Bainbridge et al., 2010). Reduced representation sequencing has been applied to anchor a large portion of the small genome (240 Mb) of woodland strawberry (Shulaev et al., 2011), but could assign only a minor fraction of the sequence assembly of the 4 Gb genome of the bread wheat progenitor *Aegilops tauschii* (Jia et al., 2013) to chromosomal locations.

**FIGURE 1:**
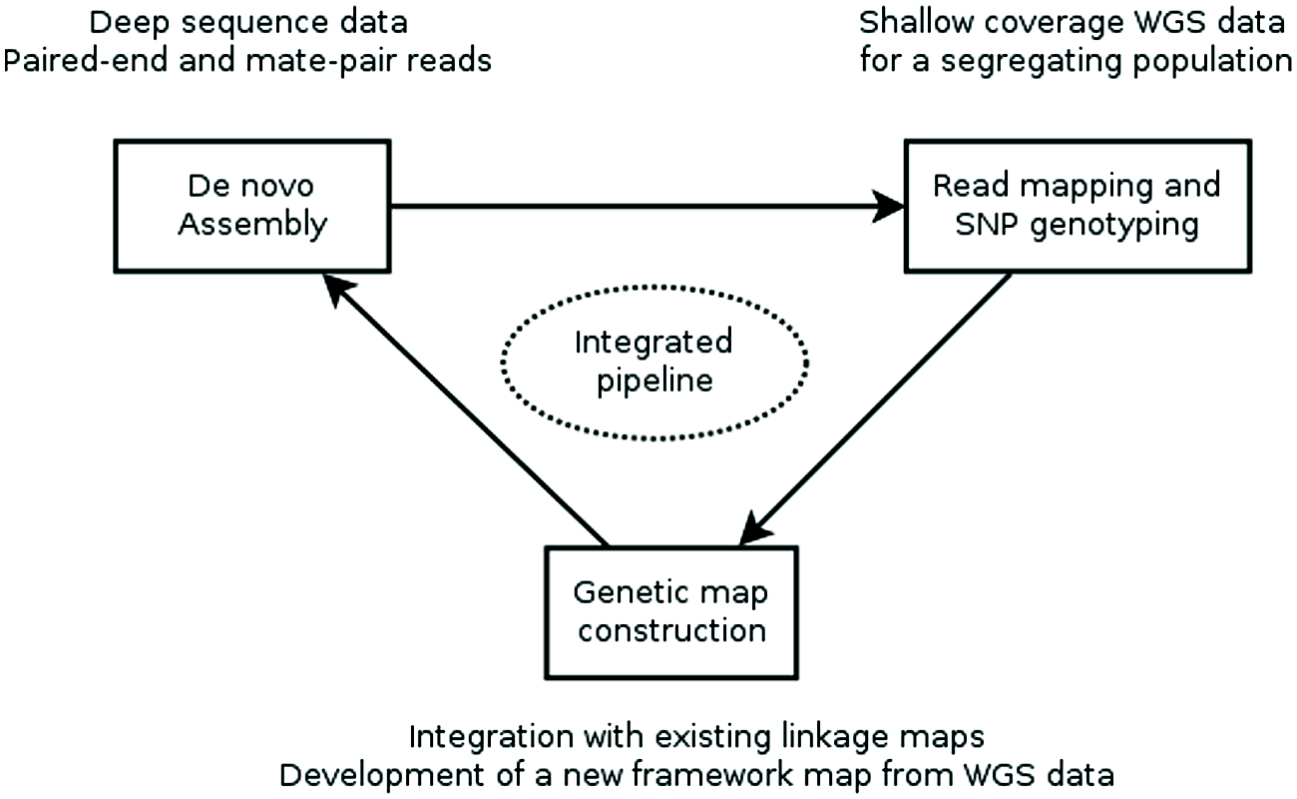
**Workflow for creating a genetically anchored WGS assembly.** A WGS assembly is created from deep sequence data of a single, ideally homozygous individual using a combination of paired-end and long-jumping mate pair reads. Shallow coverage sequence data from the individuals of a genetic mapping population is mapped to this assembly and SNPs are detected and genotyped *in silico* in the population. This genotypic data is used to construct a linkage map or is integrated with existing maps. Finally, contigs are placed to the genetic map based on the segregation patterns of the SNP markers they carry. Currently, assembly, SNP genotyping and genetic map construction are performed consecutively with existing general-purpose tools. Future research should focus on developing an integrated pipeline, where an initial assembly is iteratively improved using sequence information from the mapping population.

Recently, two reports (Mascher et al., 2013; Hahn et al., 2014) described computational pipelines that employ genotyping by whole genome sequencing of a genetic mapping population to construct an ultra-dense *de novo* linkage map of this population and place the assembly contigs of a WGS assembly into the map, producing a genetically anchored WGS assembly. The major computational steps of these procedures are: (i) constructing a WGS assembly from NGS data, (ii) mapping the sequence reads of the population to the assembly and computational genotype calling, (iii) building a genetic linkage map as a framework into which to (iv) integrate the WGS SNPs and assembly contigs harboring them (**Figure 1**).

The POPSEQ method (Mascher et al., 2013) utilizes established software for read mapping (BWA (Li and Durbin, 2009), variant calling [SAMtools (Li, 2011)] and map-making [MSTMap (Wu et al., 2008)]. SNPs detected by whole-genome sequencing of a mapping population are placed into a genetic framework of this same population through a simple nearest neighbor search. POPSEQ was first used to anchor genetically an existing genome assembly of barley (*Hordeum vulgare*), a monocotyledonous crop plant. The individuals of two mapping populations were sequenced to average onefold whole-genome coverage and after *in silico* genotyping, SNPs were placed into genetic framework maps of the populations which had been previously constructed from SNP array data (Comadran et al., 2012), through genotyping-by-sequencing (Poland et al., 2012), or were made from the WGS data of the population. The genetic positions of SNPs on WGS contigs were then used to assign chromosomal locations to the contigs of the WGS assembly. Two thirds (1.2 Gbp) of the 1.8 Gbp barley assembly could thus be genetically localized. Although the anchored portion of the assembly included 80% of the predicted gene loci, the assembly itself represented only the low-copy portion of the large (5 Gb) and highly repetitive barley genome (The International Barley Genome Sequencing Consortium, 2012).

A similar method [recombinant population genome construction, RPGC(Hahn et al., 2014)] likewise combines existing tools for sequence-based genotyping [BWA (Li and Durbin, 2009), SAMtools (Li, 2011), GATK (DePristo et al., 2011)] and genetic map construction [MSTMap (Wu et al., 2008)]. An additional feature of RPGC is the detection and correction of assembly errors caused by erroneously collapsing highly similar paralogous sequences. Such collapsed loci show segregation patterns inconsistent with a 1:2:1 distribution of genotypes in an F_2_ population. The authors evaluated RPGC with simulated sequence data of an F_2_ population of the worm *C. elegans*, a model species with a small genome (∼100 Mb). A *de novo* assembly with ALLPATHS-LG (Gnerre et al., 2011) consisted of only 88 scaffolds and covered 96% of the genome. Alignment to the *C. elegans* reference genome revealed that all scaffolds were ordered and oriented correctly, indicating that NGS-based sequence assembly and subsequent anchoring may be able to create almost complete and highly accurate sequence assemblies for species with small, repeat-poor genomes.

POPSEQ and RPGC are both targeted towards the construction of a reference sequence for a given species. Nevertheless, the availability of a reference genome does not at all depreciate further *de novo* assembly efforts. Structural variation is abundant in the genomes of many species (Feuk et al., 2006; Springer et al., 2009; Munoz-Amatriain et al., 2013; Marroni et al., 2014). Because complex events resulting in copy-number or presence absence variation are difficult to disentangle by mapping short NGS reads to a single reference sequence (Medvedev et al., 2009; Alkan et al., 2011a), reference-guided *de novo* assembly (Schneeberger et al., 2011) has been proposed as a tool to detect large-scale deletions, insertions and inversions. In a recent example, Gao et al. (2013) used sequence data from a segregating population of rice to assemble the genome sequence of one parent and correct errors in the existing assembly of the other parent.

Anchoring sequence scaffolds by population sequencing can also benefit on-going map-based sequencing projects. Although the construction of genetically anchored WGS assemblies is independent of a physical map and associated sequence resources (sequenced BAC clones, BAC end sequences), both can synergistically improve each other. As shown for barley, the sequence and marker resources provided by the assembly can be used to order and anchor the physical map (Ariyadasa et al., 2014) and, vice versa, the information about short-range connectivity obtained from clone overlaps can help further resolve the order of sequence contigs within recombination bins.

## APPLICATIONS OF GENETICALLY ORDERED SEQUENCE ASSEMBLIES

The genome sequence of a species is not an end in itself. But a genome constitutes a “research infrastructure” for biology (Olson, 1993), providing a stepping stone to a wide range of studies in basic and applied research that either makes possible or greatly accelerates the achievement of their aims. Many of these applications do not strictly necessitate a finished reference genome, i.e., near-complete pseudomolecules for each chromosome, but they can also be carried out with a partially ordered sequence assembly (possibly supplemented by physical mapping resources) that represents the majority of gene models. Such a partial order can be provided by genetically ordered WGS assemblies, which may function as hubs for gene isolation and empower comparative and evolutionary genomics.

**FIGURE 2:**
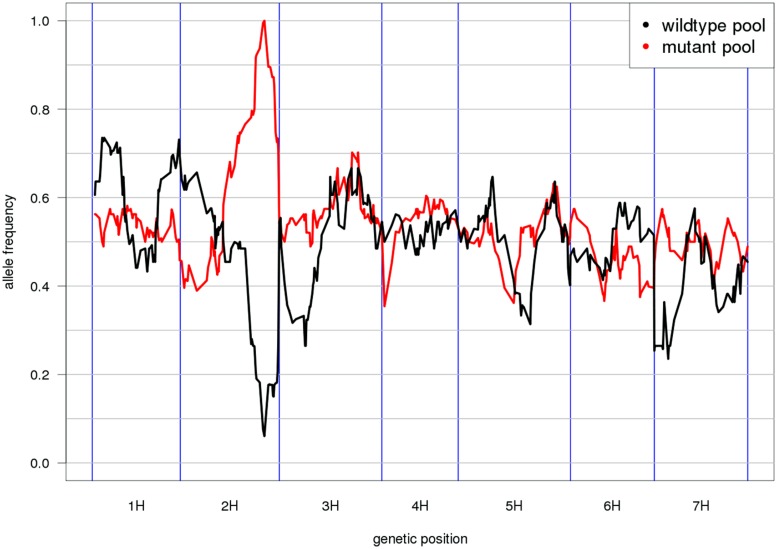
**Reference-based genetic mapping.** A genetically ordered gene-space assembly can function as an effective surrogate for a reference genome sequence for the purpose of mapping-by-sequencing (Schneeberger et al., 2009). This example shows the allele frequency distribution along the POPSEQ map of barley (Mascher et al., 2013) in two contrasting bulks of the Oregon Wolfe Barley (OWB) population. The bulks were defined by presence or absence of the *zeocriton* (compressed ear) phenotype. Eighty-two OWB individuals were sequenced as part of POPSEQ and phenotypes were assigned based on publicly available phenotypic data (Carollo et al., 2005). The peak coincides with the genetic position of the gene *ZEOCRITON* (2H, 127 cM; Houston et al., 2013).

Mapping-by-sequencing is the combined use of bulked segregant analysis and NGS to identify genes that underlie phenotypic traits (Schneeberger and Weigel, 2011). After the initial implementation in Arabidopsis (Schneeberger et al., 2009), similar approaches have been developed in other plant and animal species (Doitsidou et al., 2010; Abe et al., 2012; Leshchiner et al., 2012). As the individuals of the mapping population are not genotyped individually but sequenced together in pools, only the distribution of allele frequencies across pools can be inspected and marker order cannot be determined *de novo*. Thus, genetic marker positions have to be inferred from an ordered reference sequence (**Figure 2**). Moreover, QTL mapping using whole-genome (Huang et al., 2009; Gao et al., 2013) or reduced representation resequencing (Baxter et al., 2011; Morris et al., 2013; Liu et al., 2014) of biparental populations or association panels can take advantage of an ordered reference to search identified target intervals for anchored candidate genes.

Genomics has been acknowledged as a powerful means to study evolutionary processes across several individuals of a single species (Luikart et al., 2003) and also across species boundaries (Sousa and Hey, 2013) to gain insights into how evolutionary forces such as adaptation to environmental conditions, natural selection, or random genetic drift shape the genomes of individuals and species. These fields have greatly benefited from the “democratization of sequencing” engendered by NGS technology (Stapley et al., 2010; Ekblom and Galindo, 2011). Genomic resources of non-model organisms can now quickly be assembled in order to support specific research aims (Ellegren, 2014). The recent study of Ellegren et al. (2012) used a genetically ordered draft genome sequence to dissect speciation between closely related songbird species. In an agronomic context, the International Oryza Map Alignment Project (Jacquemin et al., 2013) aims at sequencing the genomes of all members of the genus *Oryza*, i.e., relatives of cultivated rice. Starting from the premise that a single reference genome is not sufficient to assess the natural diversity across an entire genus, this project wants to establish a comprehensive genomic infrastructure to empower studies into the evolutionary dynamics of genome structure, conservation genomics and to assist crop improvement by introgressing beneficial alleles into elite germplasm. This endeavor could probably benefit from population sequencing data to anchor WGS assemblies and physical maps.

## CHALLENGES AND LIMITATIONS

The most time-consuming step of anchoring a WGS assembly is the construction of a genetic population. While the sequencing and computational steps can be carried out in less than 6 months (Mascher et al., 2013), population development, in case of recombinant inbred line populations, may involve several rounds of self-fertilization, which can take several years. However, in plants, genetic mapping is routinely performed by researchers in academia and private industries and suitable mapping populations are often readily available. Moreover, plant mapping populations lend themselves very well to sequence-based mapping. Populations are generally started from highly homozygous genotypes and advanced recombinant inbred or doubled haploid progeny lines are nearly or completely homozygous, respectively. By contrast, F_2_ generations involve only one round of selfing after the initial cross. However, half of the genome of F_2_ individuals is expected to be heterozygous, requiring deeper sequence coverage for reliable genotyping. Even in obligate outcrossers, linkage maps can be made from crosses between heterozygous parents (Grattapaglia and Sederoff, 1994). Although controlled crosses cannot be made and the progeny of a single pair of parents is limited in number, genetic mapping is anything but impossible in animals. Linkage analysis in families of siblings from a cross between heterozygous parents is more complicated than in the progeny of homozygous lines (Maliepaard et al., 1997). Markers differ in the number of alleles and the number of heterozygous parents, and it can be impossible to determine the linkage phase of a marker, i.e., from which grandparent it was inherited. High-density linkage maps of the human genome have been constructed from multi-generation pedigrees (Dib et al., 1996). Similar methods based on three-generation pedigrees have been applied in other mammalian species such as macaque monkeys (Rogers et al., 2006) and domestic cats (Menotti-Raymond et al., 1999). Moreover, RIL populations have been created by mating of full-siblings in the laboratory animals mouse (Williams et al., 2001), rat (Pravenec et al., 1996), and fruit fly (Nuzhdin et al., 1997). If a robust genetic framework map can be computed, whole genome sequencing of the pedigree should allow populating this framework with additional markers and WGS sequence contigs. The heterozygosity of natural pedigrees may necessitate deeper sequencing to reliably score heterozygotes. Recent studies found that genotype calling from low- or medium-coverage (<15x) data often results in calling heterozygotes as homozygotes and can bias downstream analyses (Kim et al., 2011; Crawford and Lazzaro, 2012). A sliding-windows approach that aggregates sequence information across multiple SNP positions may help mitigate the effects of genotyping errors caused by low read depth.

In any species, linkage mapping has the inherent limitation that the maximally achievable resolution is determined by the recombination landscape, or more specifically, the ratio between physical and genetic distance along the genome. In grasses, for example, recombination events mainly occur in distal regions, whereas large peri-centromeric intervals are almost devoid of cross-overs. These so-called genetic centromeres correspond to a single large bin in a genetic map, which can only be resolved with extremely large mapping populations or possibly through alternative approaches such as physical mapping (van Oeveren et al., 2011), optical mapping (Dong et al., 2013), or methods based on chromosomal conformation capture (Lieberman-Aiden et al., 2009; Burton et al., 2013).

In contrast to these intrinsic difficulties given by biological facts, algorithmic parameters of the anchoring process can be subject to directed improvement. The major computational tasks of assembly anchoring are *de novo* assembly, read mapping, variant calling and linkage map construction. One of the major determinants of anchoring efficiency is assembly contiguity. The longer a sequence contig is, the more likely it is that at least one sequence polymorphism can be detected to anchor it. Furthermore, longer contigs alleviate the problem of missing data. Even though the majority of individuals have missing genotype calls for single SNPs as a consequence of shallow-coverage sequencing (Huang et al., 2009; Mascher et al., 2013), aggregating genotypic information across all SNPs on a single contig results in consensus genotype calls with little or no missing data.

In the approaches of Mascher et al. (2013) and Hahn et al. (2014), read mapping and variant calling are performed with standard tools that are routinely used in large-scale resequencing projects (1000 Genomes Project Consortium et al., 2012; Tennessen et al., 2012) and will likely scale with the growing amount of raw data as population size and sequencing depth increase. By contrast, the majority of genetic mapping programs are still tailored to datasets encompassing only a few 1000 markers. The most commonly used tool to compute linkage maps form larger marker sets is MSTMap (Wu et al., 2008), for which excessive runtimes have been reported when marker order exceeds ∼100,000 (Howe et al., 2013). As the number of recombination bins in small biparental populations is limited, it can be envisaged to cluster markers prior to map-making based on their segregation patterns to obtain a smaller, yet fully informative set of framework markers. Moreover, focusing on a small number of high-confidence SNP loci may avoid the common problem of map-inflation, which is often caused by spurious cross-over events introduced by genotyping errors (Cheema and Dicks, 2009).

Moreover, valuable insights into the choice of parameters, the overall accuracy of the methods and the interplay of sequencing depth, population size, and final mapping resolution may be gained by performing *de novo* assembly and anchoring on real data gathered from species with existing high-quality reference genomes to be used as a gold standard for benchmarking.

## CONCLUSION

The interest in the genome sequencing of non-model species (Ellegren, 2014) or economically important species with humongous genomes (Brenchley et al., 2012; Nystedt et al., 2013) has increased recently. Genome sequencing and assembly efforts and novel algorithmic development can be expected to intensify in the years to come, as genome sequencing of thousands of animal and plant species has been proposed (Genome 10K Community of Scientists, 2009; Johnson et al., 2012). We conclude with reiterating the advice of Gao et al. (2013) that each genome assembly project should, if at all possible, obtain WGS data from at least one segregating population. Short read assembly will remain a central part of any genome project as long as advances in sequencing technology will not make possible chromosome-sized sequence scaffolds. In the meantime, methods such as genetic anchoring will always be necessary to enhance the utility of fragmented WGS assemblies.

## Conflict of Interest Statement

The authors declare that the research was conducted in the absence of any commercial or financial relationships that could be construed as a potential conflict of interest.
